# Supplementary material for: Lysine 68 acetylation directs MnSOD as a tetrameric detoxification complex versus a monomeric tumor promoter
Source: Nat Commun. 2019 Jun 3;10:2399. doi: 10.1038/s41467-019-10352-4 (PMC6546705; doi:10.1038/s41467-019-10352-4)
Supplement: Supplementary file 1 — Supplementary Information [file 41467_2019_10352_MOESM1_ESM.pdf]

SUPPLEMENTARY INFORMATION

Lysine 68 acetylation directs MnSOD as a tetrameric detoxification complex versus a monomeric tumor promoter

Yueming Zhu, Xianghui Zou et al.

Supplementary Note 1. Supplementary Figure Inventory

Figure 1a relates to manuscript figure 1b.
Figure 1b relates to manuscript figure 2a,b.

Figure 2a relates to manuscript figure 2c.
Figure 2b relates to manuscript figure 2d.
Figure 2c relates to manuscript figure 2c.
Figure 2d relates to manuscript figure 2c.
Figure 2e relates to manuscript figure 2c.
Figure 2f relates to manuscript figure 2c.

Figure 3a relates to manuscript figure 3e.
Figure 3b relates to manuscript figure 3e.
Figure 3c relates to manuscript figure 3e.
Figure 3d relates to manuscript figure 3e.

Figure 4a relates to manuscript figure 4.
Figure 4b relates to manuscript figure 4.
Figure 4c relates to manuscript figure 4.

Figure 5a relates to manuscript figure 4e.
Figure 5b relates to manuscript figure 4e.
Figure 5c relates to manuscript figure 4e.
Figure 5d relates to manuscript figure 4f.
Figure 5e relates to manuscript figure 4f.
Figure 5f relates to manuscript figure 4f.

Figure 6a relates to manuscript figure 6a.
Figure 6b relates to manuscript figure 6b.
Figure 6c relates to manuscript figure 6c.
Figure 6d relates to manuscript figure 6d.
Figure 6e relates to manuscript figure 6f.
Figure 6f relates to manuscript figure 6g.
Figure 6g relates to manuscript figure 6h.

Figure 7a relates to manuscript figure 7a.
Figure 7b relates to manuscript figure 7d.
Figure 7c relates to manuscript figure 7f.
Figure 7d relates to manuscript figure 7g.
Figure 7e relates to manuscript figure 7h.

Figure 8a relates to manuscript figure 7i.
Figure 8b relates to manuscript figure 7.
Figure 8c relates to manuscript figure 7.
Figure 8d relates to manuscript figure 7.
Figure 8e relates to manuscript figure 7.
Figure 8f relates to manuscript figure 7.
Figure 8g relates to manuscript figure 7.

Figure 9a relates to manuscript figure 7.
Figure 9b relates to manuscript figure 7.
Figure 9c relates to manuscript figure 7.
Figure 9d relates to manuscript figure 7.
Figure 9e relates to manuscript figure 8a.

Figure 10a relates to manuscript figure 8.
Figure 10b relates to manuscript figure 8.
Figure 10c relates to manuscript figure 8.
Figure 10d relates to manuscript figure 8.

Figure 11a relates to the discussion.
Figure 11b relates to the discussion.

a

pMEFs (lenti-Myc)	Doubling Time	Xenograft growth
Control	31h	-
MnSOD^{K68R}	68h	-
MnSOD^{K68Q}	25h	↑↑↑

b

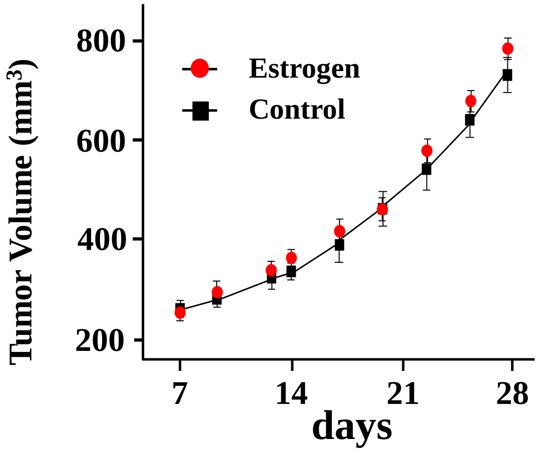

Supplementary Figure 1. Expression of *MnSOD^{K68Q}* decreased doubling time, allowed xenograft growth, and caused estrogen independence. (a) pMEFs were infected with lenti-Myc (control) and either lenti-MnSOD^{K68R} or lenti-MnSOD^{K68Q}. Cells were selected in puromycin for 14 Days; then medium was replaced every 2 days for 28 days, and cell growth rate was evaluated. Doubling time for the pMEFs-control, pMEF-Myc-MnSOD^{K68R}, and pMEF-Myc-MnSOD^{K68Q} cells (middle column) was determined by Td=(t2-t1)*log(2)/log(q2/q1). The MCF7 cells infected with lenti-Myc (control) and either lenti-MnSOD^{K68R} or lenti-MnSOD^{K68Q} were also used for xenograft growth experiments where 1 million cells were implanted into both hind limbs of nude mice. The tumor volumes were measured every 3 days. The control and Myc-MnSOD^{K68R} cells did not form tumors while the Myc-MnSOD^{K68Q} cells formed xenograft tumors. **(b)** MCF7 cells infected with lenti-MnSOD^{K68Q}, and selected in puromycin for 14 days, were subsequently implanted into both hind limbs of nude mice without (black squares) or with estrogen supplementation (red circles). The tumor volumes were measured every 7 days (1.0 x 10^6 cells). Three upward arrows represent that all 10 nude mice hind leg infections grew xenografts. All experiments were done in triplicate. Error bars represent ± 1 SEM.

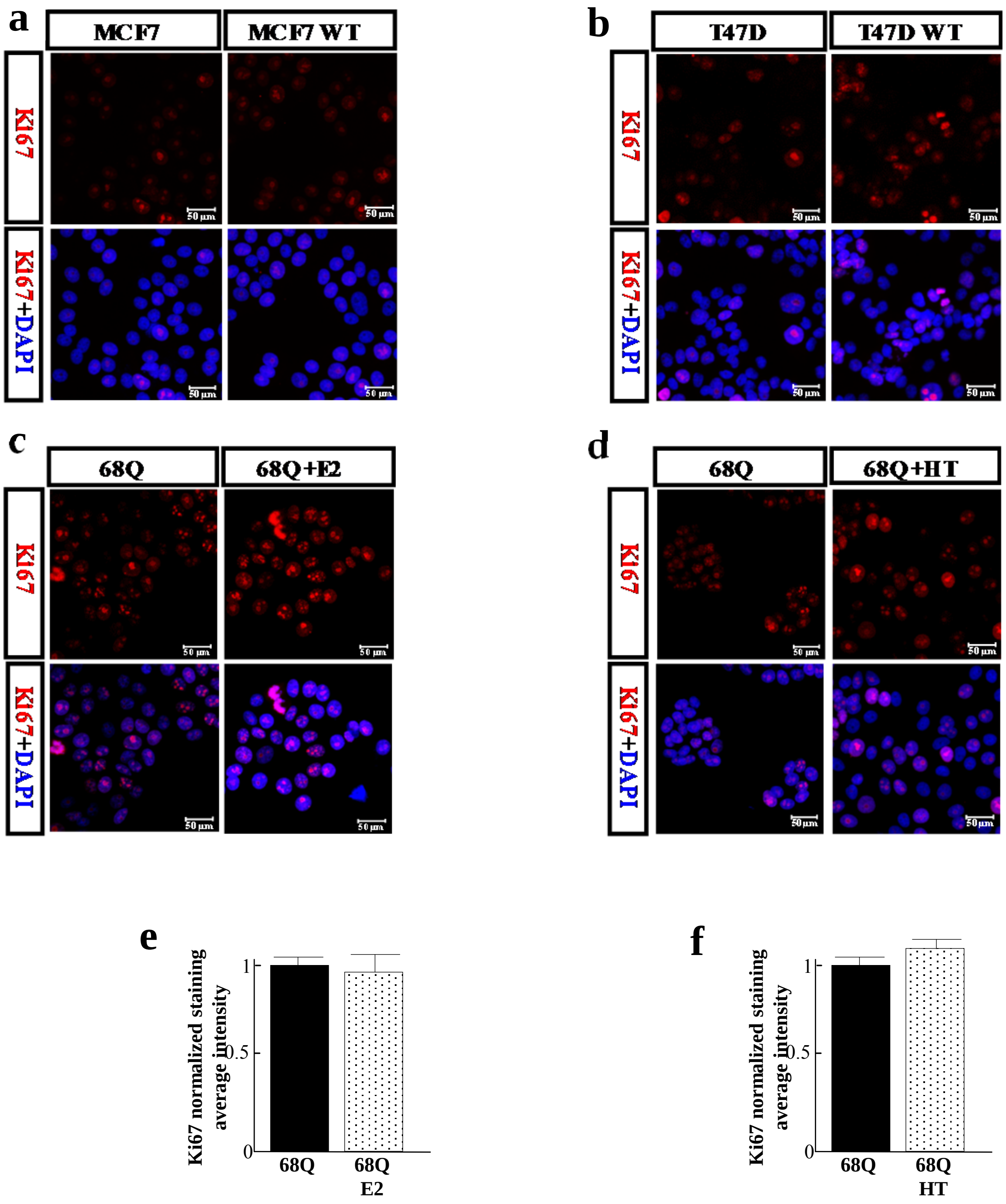

Supplementary Figure 2. (a,b) MCF7 cells and MCF7-MnSOD^{WT} cells stained for Ki-67 levels. Asynchronously growing cultures of **(a)** MCF7 and MCF7-MnSOD^{WT} cells, as well as **(b)** T47D and T47D-MnSOD^{WT} cells, constructed by infection with lenti-MnSOD^{WT} or the empty control lentivirus. After 24 h of growth on glass coverslips, cells were fixed and stained with anti-Ki-67 and anti-DAPI antibodies. **(c,d) MCF7-MnSOD^{K68Q} cells exposed to either estrogen or Tam, and stained for Ki-67.** MCF7-MnSOD^{WK68QT} cells were exposed to either **(c)** estrogen (E2) for 5 days or **(d)** 1 μM 4-hydroxy-Tam (HT) for 5 days. Cells were replated on glass coverslips for 24 h with same concentrations of E2 or HT. Cells were then fixed and subsequently stained with anti-Ki-67 and anti-DAPI antibodies. **(e-f)** Quantifications of average Ki-67 intensity in panels (c) and (d) are shown in the bar graphs. All experiments were done in triplicate. Error bars represent ± 1 SEM. Representative IHC images are shown.

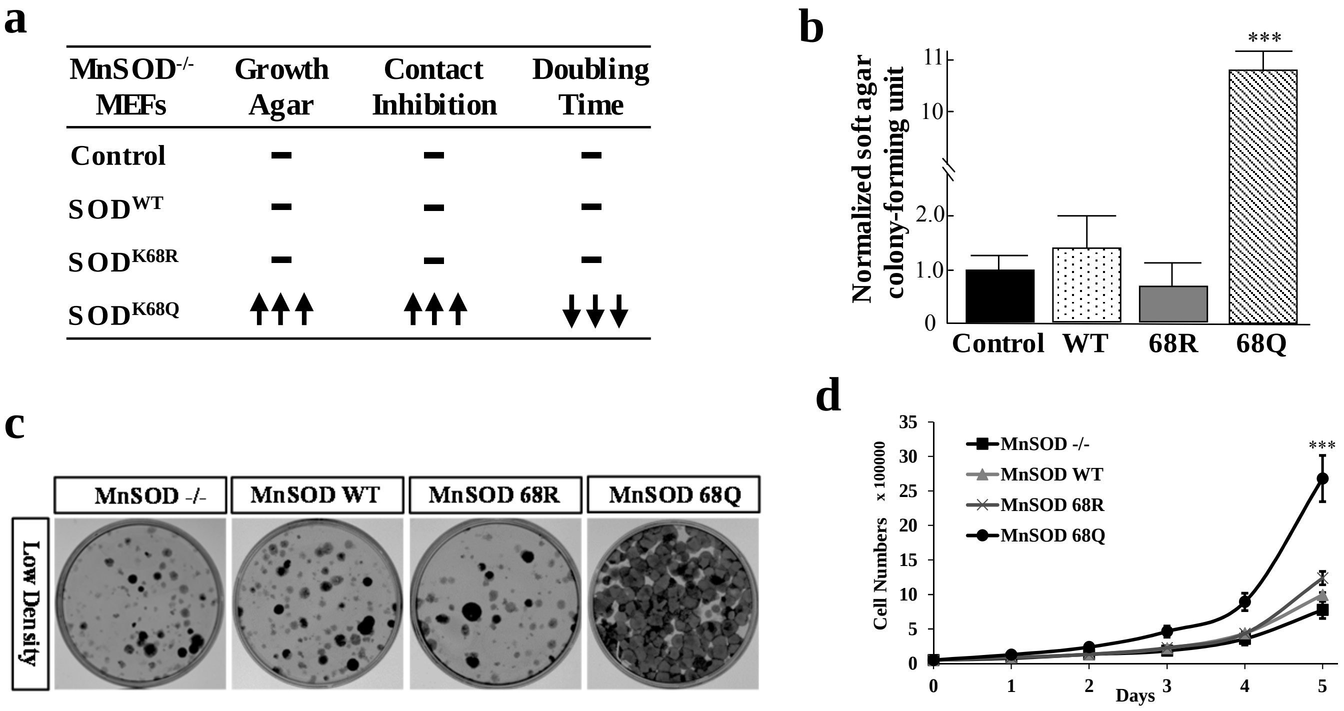

Supplementary Figure 3. *MnSOD^{K68Q}* expression promotes a transformation-permissive phenotype *in vitro*. (a) MnSOD$^{-/-}$ MEFs were infected with lenti-MnSOD^{WT}, lenti-MnSOD^{K68R}, and lenti-MnSOD^{K68Q} and cells were cultured and selected in puromycin for 14 days. The MnSOD$^{-/-}$ MEFs expressing *MnSOD^{K68Q}* exhibited a more transformed phenotype, as compared to either cells expressing *MnSOD^{K68R}* or *MnSOD^{WT}*, well as non-infected cells (MnSOD$^{-/-}$). **(b)** 100 or 250 cells from all four of these cell lines were plated per 60 mm dish, and after 14 days cells were stained with crystal violet to determine the growth at low density. **(c)** 10,000 cells from all four of these cell lines were plated on 0.3% agar over 0.6% base agar for 21 days, and colonies were counted. **(d)** 20,000 cells from all four of these cell lines were plated per 60 mm dish and measured each day, and doubling time was determined by Td=(t2-t1)*log(2)/log(q2/q1). All experiments were done in triplicate. Error bars represent ± 1 SEM. ***p < 0.001. Representative images are shown.

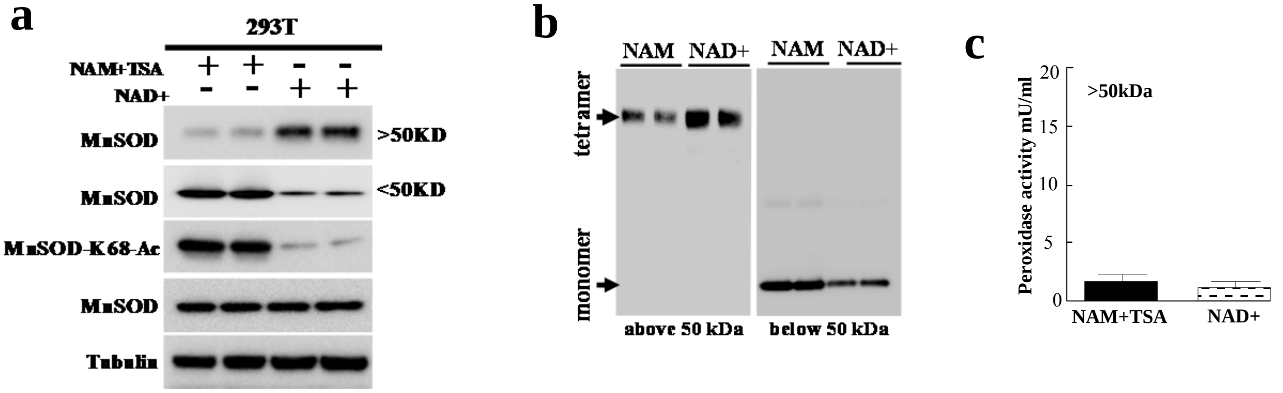

Supplementary Figure 4. (a-c) The physical acetylation of MnSOD-K68 produces peroxidase activity. (a) 293T cells were transfected with plasmids expressing *Flag-MnSOD*^{WT} and treated with either 10 mM NAM and 1 μM TSA, or 10 mM NAD+, harvested at 40 h, and IPed with anti-Flag antibody. The IPed samples were separated using 50 kDa centrifugal filters and protein extracts above and below 50 kDa were isolated, followed by immunoblotting with anti-MnSOD, MnSOD-K68-Ac, and actin antibodies. **(b)** The samples expressing *Flag-MnSOD*^{WT} and treated with 10 mM NAM and 1 μM TSA or 10 mM NAD+ were separated using 50 kDa centrifugal filters. Samples were subsequently run on a semi-native gel and immunoblotted with an anti-MnSOD antibody. **(c)** MnSOD$^{-/-}$ immortalized MEFs were transfected with plasmids expressing Flag-MnSOD^{WT} and treated with either 10 mM NAM and 1 μM TSA, or 10 mM NAD+, and cells were harvested at 40 h and IPed with anti-Flag antibody. The IPed samples were separated using 50 kDa centrifugal filters and protein exacts above 50 kDa were isolated, and purified proteins were then used for biochemical analysis of peroxidase activity. All experiments were done in triplicate. Error bars represent ± 1 SEM. Representative images are shown.

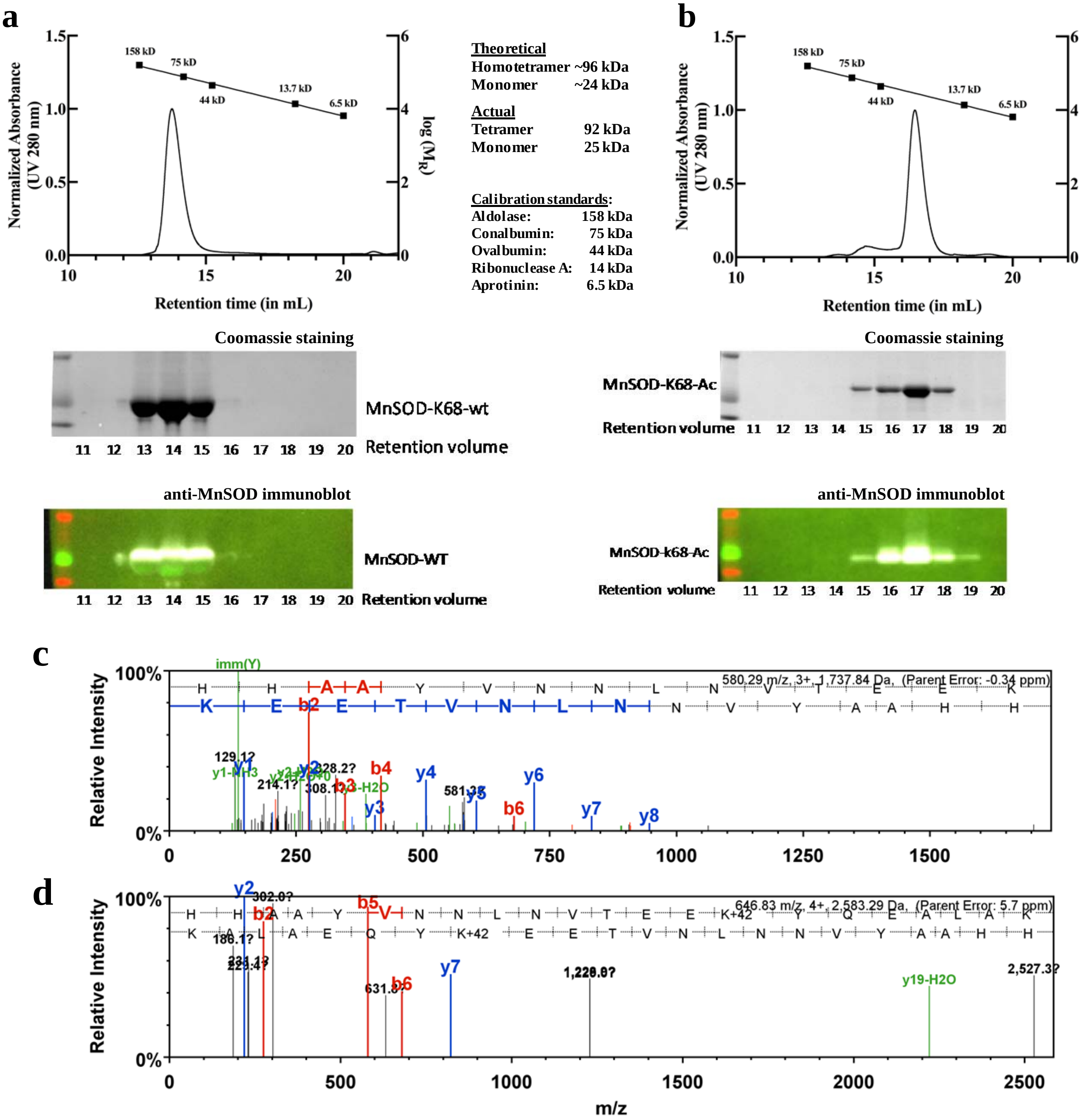

e

Gene Name	Protein Identification probability	Number of unique peptides	Number of unique spectra	Number of total spectra	Percentage Lysine 68 acetylation
pET21a-MnSOD^{WT}	100%	32	164	999	9.9%
pET21a-MnSOD^{K68TAG}	100%	24	99	531	80.1%

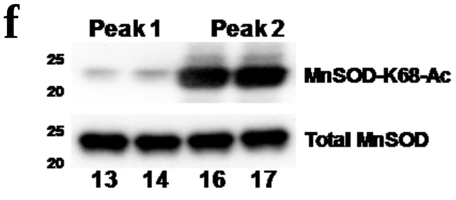

Supplementary Figure 5. MnSOD-K68 acetylation exhibits peroxidase activity. BL21(DE3) bacteria were transformed with pET21a-MnSOD^{WT}, or pEVOL-AcKRS together with pET21a-MnSOD^{K68TAG}. Cells were harvested and lysed, and eluted protein were run over a Superdex 200

Increase 10/300 GL column and fractions[1, 2, 3], and these samples were subsequently used for further analysis. **(a)** Chromatogram from the size exclusion column of purified protein from bacteria carrying pET21a-MnSOD^{WT} (top panel), retention volumes fractions 11 through 20 were further analyzed by either Coomassie staining (middle panel) or immunoblotted with anti-MnSOD antibody (lower panel) to confirm MnSOD levels. **(b)** Chromatogram of purified protein from bacteria carrying pEVOL-AcKRS and pET21a-MnSOD^{K68TAG}, all the fractions were further analyzed by either Coomassie staining (middle panel) or immunoblotted with an anti-MnSOD-K68-Ac antibody (lower panel) The raw data are presented with the y-axis as mAU (280 nm) to show that peak 2 is smaller than peak 1 which is likely due to the slightly less protein run on the Superdex 200 Increase 10/300 GL column (5.5 mg vs. 4.8 mg). **(c)** Three separate MnSOD-K68-WT samples were analyzed via mass spectroscopy and 32 exclusive unique peptides, 164 spectra, and 999 total spectra, 100% coverage which is an average of each run. **(d)** Three separate MnSOD-K68-Ac samples showed 24 exclusive unique peptides, 99 unique spectra, 531 total spectra were identified, 95% coverage which is an average of each run. **(e)** Table showing the average percentage of total number of unique K68 acetylated peptides, as a ratio of the total number of unique peptides. The data for total number of unique peptides, unique spectra, and total spectra from bacteria expressing pET21a-MnSOD^{WT} or expressing pET21a-MnSOD^{K68TAG} are also shown. **(f)** Peak 1 (volumes 13, 14 ml) and peak 2 (volumes 16, 17 ml) were separated by SDS-PAGE and immunoblotted with anti-MnSOD-K68-Ac antibody. All experiments done in triplicate. Representative images are shown.

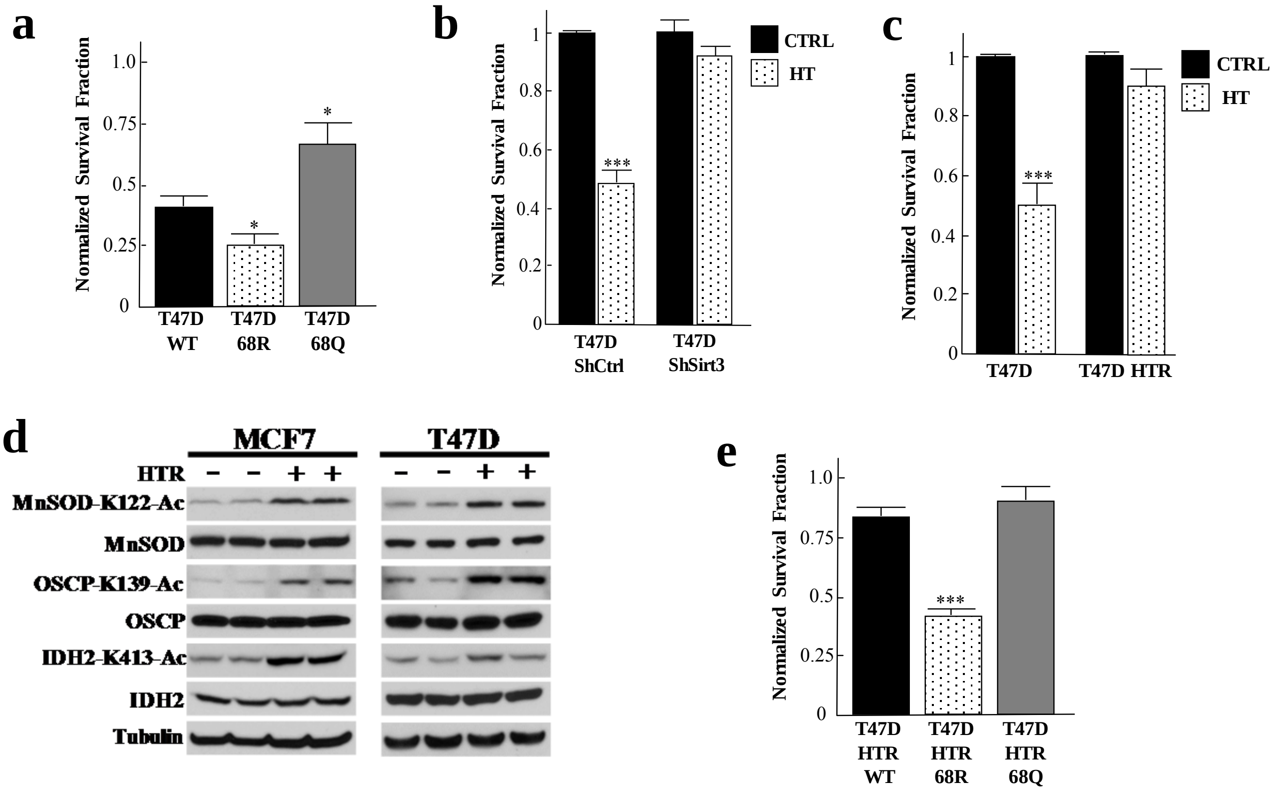

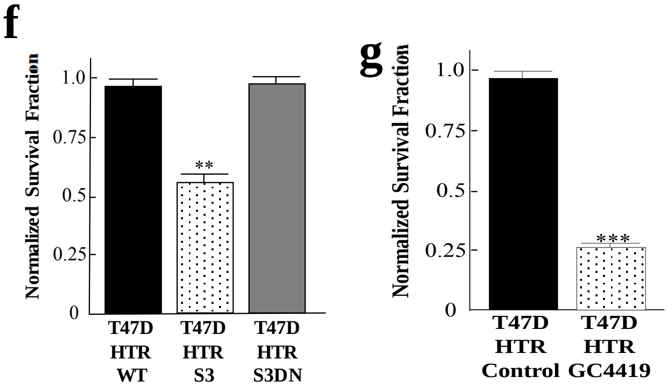

Supplementary Figure 6. Loss of SIRT3-induced MnSOD-K68 deacetylation leads to hydroxy-Tam resistance in human breast cancer cells. (a) T47D-MnSOD^{WT}, T47D-MnSOD^{K68R}, and T47D-MnSOD^{K68Q} permanent cell lines were selected for hydroxy-Tam resistance in 1 µM for 3 months, and clonogenic cell survival experiments were completed. **(b)** T47D-shCtrl and T47D-shSIRT3 permanent cell lines were exposed to 1 µM 4-hydroxy-Tam For 24 h (HT), and clonogenic cell survival experiments were done. **(c)** T47D and T47D-HTR cells, with and without exposure to 1 µM 4-hydroxy-Tam for 24 h (HT), were measured for cytotoxicity by clonogenic survival experiments. **(d)** MCF7 cells (left), and T47D cells (right) were cultured in regular DMEM containing 1 µM hydroxy-Tam for 3 months (HT). The cell lysates were analyzed by immunoblotting with anti-MnSOD-K122-Ac (validated as a SIRT3 deacetylation target in Tao et al., 2010, *Cancer Cell*), anti-MnSOD, anti-OSCP-K139-Ac (validated as a SIRT3 deacetylation target in Tao et al., 2010, *Cancer Cell*), anti-OSCP, anti-IDH_2K413-Ac (validated as a SIRT3 deacetylation target in Someya et al., 2010, *Cancer Cell*), anti-IDH_2, and anti-actin. **(e)** T47D-HTR cells were infected with lenti-MnSOD^{WT}, lenti-MnSOD^{K68Q}, or lenti-MnSOD^{K68R} and treated with 1 µM 4-HT for 24 h, followed by clonogenic cell survival assays. **(f)** T47D-HTR cells were infected with lenti-SIRT^{WT} (S3) or lenti-SIRT^{DN} (S3DN; dominant-negative deacetylation-null gene) and treated with 1 µM hydroxy-Tam for 24 h, followed by clonogenic cell survival assays. **(g)** T47D-HTR cells were incubated with 5 µM GC4419 for 5 days, followed by clonogenic cell survival assays. All experiments were done in triplicate. Error bars represent ± 1 SEM. *$p < 0.05$, **$p < 0.01$, and ***$p < 0.001$.

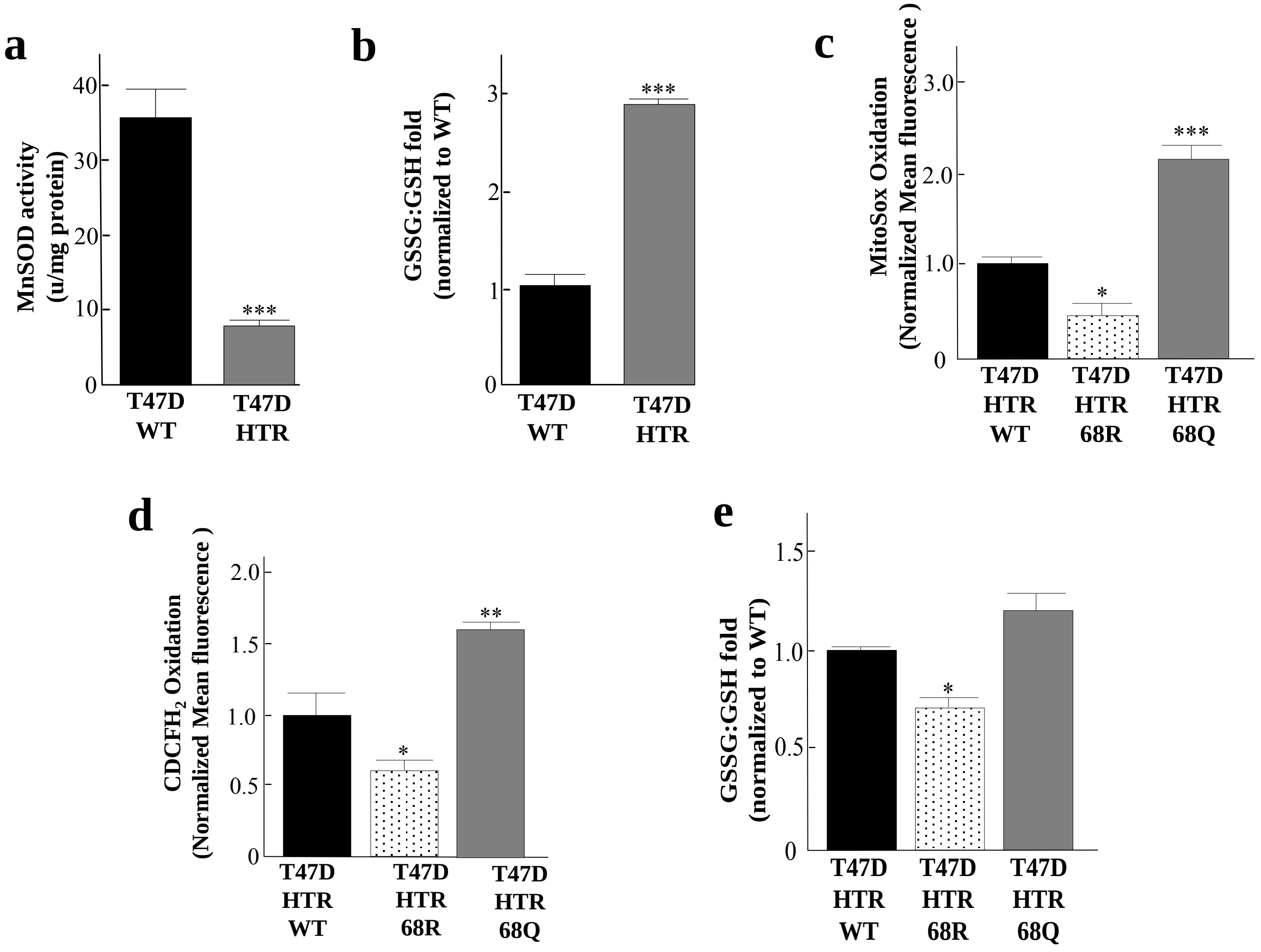

Supplementary Figure 7. The increased oxidative stress in hydroxy-Tam resistant human breast cancer cells can be reversed by expression of MnSOD^{K68R}. (a,b) T47D cells selected for 3 months in 1 μM hydroxy-Tam were harvested, and whole-cell homogenates were used for: **(a)** biochemical analysis of total MnSOD activity and **(b)** biochemical analysis of glutathione levels. **(c-e)** T47D-HTR cells were infected with lenti-MnSOD^{WT}, lenti-MnSOD^{K68R}, or lenti-MnSOD^{K68Q} and harvested. Whole-cell homogenates were used for **(c)** biochemical analysis of MitoSox oxidation; **(d)** biochemical analysis of H_2O_2 as detected by $CDCFH_2$ oxidation; and **(e)** biochemical analysis of glutathione levels. All experiments were done in triplicate. Error bars represent ± 1 SEM. *p < 0.05, **p < 0.01, and ***p < 0.001.

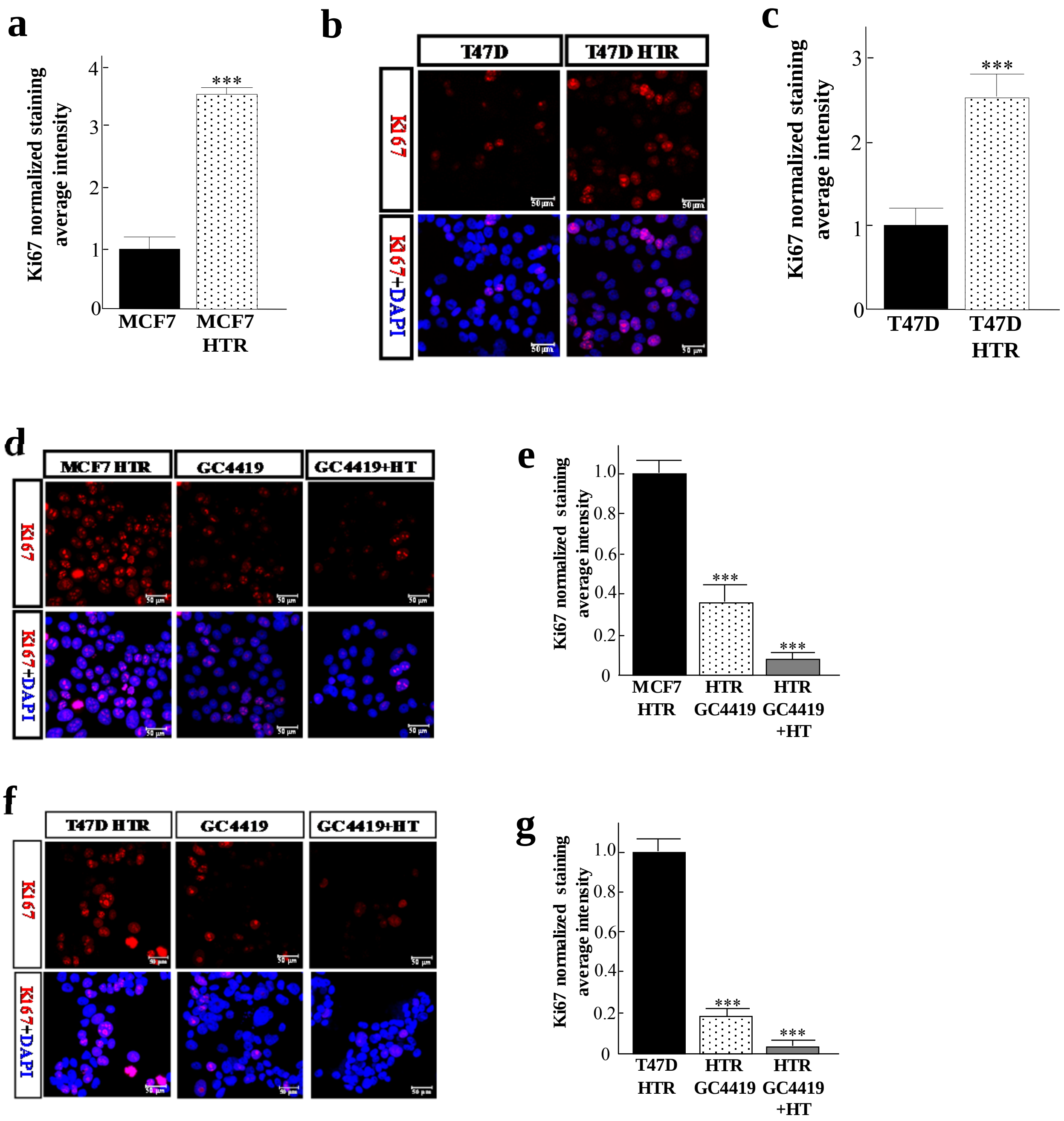

Supplementary Figure 8. MnSOD mimetic GC4419 decreased Ki-67 levels in T47D-HTR cells. (a) The data from Figure 7i, where MCF7-HTR cells were stained for Ki-67, as well as DAPI, were counted with ImageJ and quantified for average Ki-67 intensity as shown in the bar graph. **(b,c)** T47D and T47D-HTR cells were stained for Ki-67 as well as DAPI, and particles in the nucleus were counted with ImageJ and quantified for average Ki-67 intensity as shown in the bar graph. MCF7-HTR **(d,e)** and T47D-HTR **(f,g)** cells were treated with 5 µM GC4419 and/or 1 µM 4-hydroxy-Tam for 5 days, and then stained for Ki-67 as well as DAPI. Quantifications of average Ki-67 intensity are shown in the bar graphs. All experiments were done in triplicate. Error bars represent ± 1 SEM. ***$p < 0.001$. Representative images are shown.

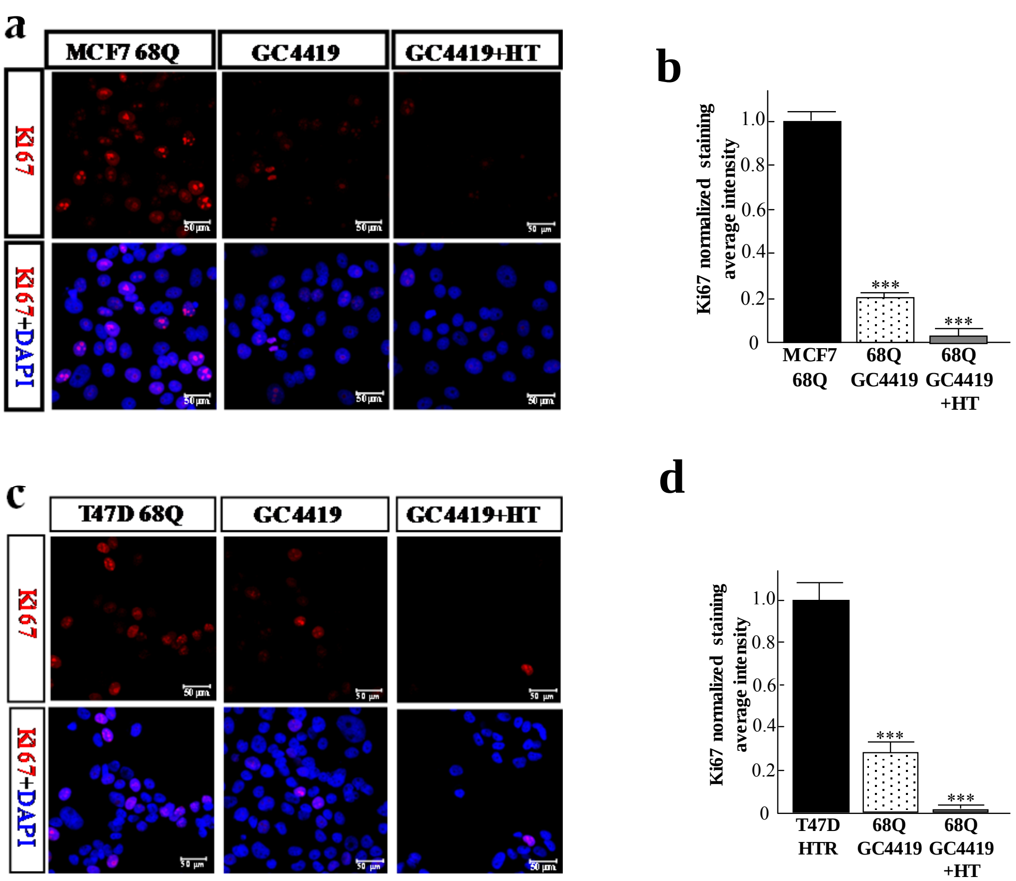

e

Group i.e., genes	Number of mice	Mice with tumor	Ave tumor weight (g)	Ave tumor size (mm^3)
MCF7	10	0	N/A	N/A
MCF7 HTR	10	10	0.71	859

Supplementary Figure 9. MnSOD mimetic GC4419 decreased Ki-67 levels in MCF7 andT47D cells expressing *MnSOD^{K68Q}*. MCF7-MnSOD^{K68Q} **(a,b)** and T47D-MnSOD^{K68Q} **(c,d)** cells were treated with 5 µM GC4419 and/or 1 µM 4-hydroxy-Tam for 5 days, and then stained for Ki-67 as well as DAPI. Quantifications of average Ki-67 intensity are shown in the bar graphs. **(e)** MCF7 and MCF7-HTR cells were implanted into both hind limbs of nude mice and tumor volumes were measured every 3 days for 6 weeks and the number of tumors that successfully implanted versus the total number of mice infected with MCF7 and MCF7-HTR as well as the average tumor weight and tumor size are shown. Representative images are shown. All experiments were done in triplicate. Error bars represent ± 1 SEM. ***$p < 0.001$.

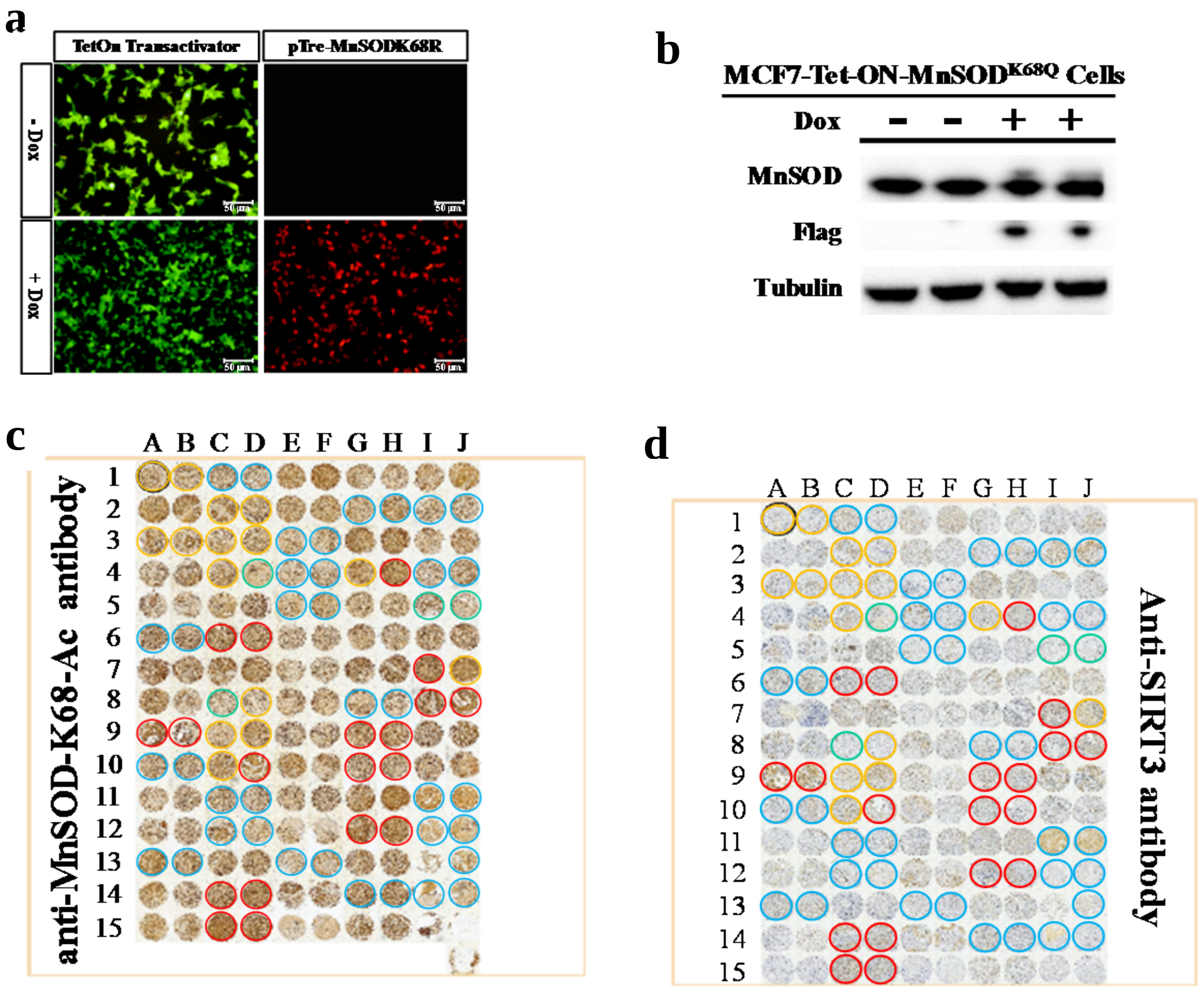

Staining Intensity	Low	Interm	High
Luminal A	26	9	2
Luminal B	4	15	19

Staining Intensity	Low	Interm	High
Luminal A	9	11	17
Luminal B	20	8	10

Supplementary Figure 10. Tet-On induced expression of MnSOD^{K68R} inhibits xenograft growth in MCF7-HTR cells. (a) MCF7-HTR cells were infected with pTet-DualOn (Clontech) and selected with puromycin followed by infection with pTre-Dual2-Flag-MnSOD^{K68R} and selection with hygromycin. These cells (MCF7-HTR-Tet-On-MnSOD^{K68Q} cells), without and with exposure to tetracycline, were tested by immunoflourescent imaging for both green (presence of pTet-DualOn) and red (presence of pTre-Dual2-Flag-MnSOD^{K68R}). **(b)** The MCF7-HTR-Tet-ON-MnSOD^{K68Q} cells above were also isolated, separated by SDS-PAGE, and immunoblotted with anti-MnSOD, Flag, and Tubulin antibodies. **A subgroup of human luminal B tumors exhibited high levels of MnSOD-K68-Ac. (c, d)** A human breast cancer TMA consisting of luminal A (n=37) and luminal B (n=38) samples that were dewaxed and immunostained with either **(c)** anti-MnSOD-K68-Ac or **(d)** anti-SIRT3 antibodies. MnSOD-K68-Ac and SIRT3 staining was grouped into low, intermediate, and high levels, and the number of samples that fell into each of these groups is presented in the table under each TMA. Red circles represent tumor samples that contain high

MnSOD-K68-Ac staining. All experiments were done in triplicate. Representative images are shown.

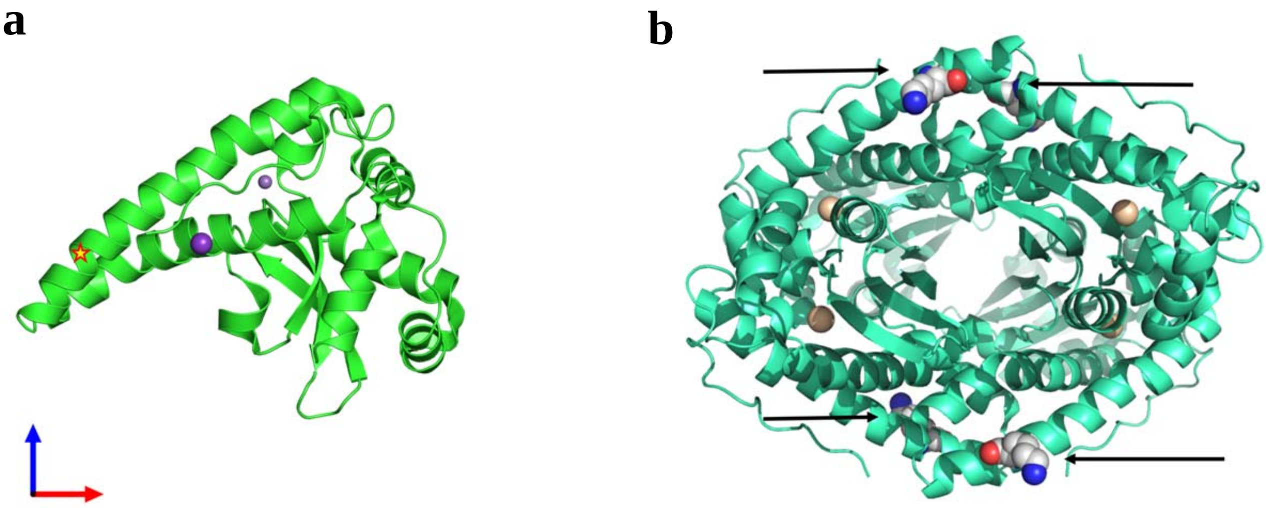

Supplemental Figure 11. Structure of MnSOD. (a) The 2.4 Å resolution structure of PDB entry 2adp rendered as a ribbon diagram. The monomeric structure of MnSOD comprises amino acids 25-222, which is the portion that forms the mature enzyme after removal of the mitochondrial targeting peptide. The position of K44 (corresponding to K68 in the full-length protein) is indicated with a yellow star. A manganese (II) ion (larger purple sphere) and a potassium ion (smaller purple sphere) are also shown. **(b)** MnSOD in tetrameric form at 1.47 Å resolution. Structure coordinates were obtained from PDB entry 1pl4. Residue K44 (K68 in the full protein) is indicated by black arrows for each monomer, with its atoms rendered as spheres and the beige spheres are manganese (II) ions.

Supplementary Information Table 1

Primer name		Primer sequence
MnSOD K68R	Forward	GTGAACAACCTGAACGTCACCGAGGAGAGGTACCAGGAGGCGTTGGCCAAG
	Reverse	CTTGGCCAACGCCTCCTGGTACCTCTCCTCGGTGACGTTCAGGTTGTTCAC
MnSOD K68Q	Forward	GTGAACAACCTGAACGTCACCGAGGAGCAGTACCAGGAGGCGTTGGCCAAG
	Reverse	CTTGGCCAACGCCTCCTGGTACTGCTCCTCGGTGACGTTCAGGTTGTTCAC
ShSIRT3	Forward	CCGGGTGGGTGCTTCAAGTGTTGTTCTCGAGAACAACACTTGAAGCACCCACTTTTTG
	Reverse	AATTCAAAAAGTGGGTGCTTCAAGTGTTGTTCTCGAGAACAACACTTGAAGCACCCAC

Supplementary References

1. Knyphausen P, *et al.* Insights into Lysine Deacetylation of Natively Folded Substrate Proteins by Sirtuins. *J Biol Chem* **291**, 14677-14694 (2016).
2. de Boor S, *et al.* Small GTP-binding protein Ran is regulated by posttranslational lysine acetylation. *Proc Natl Acad Sci U S A* **112**, E3679-3688 (2015).
3. Lammers M. Expression and Purification of Site-Specifically Lysine-Acetylated and Natively-Folded Proteins for Biophysical Investigations. *Methods in molecular biology (Clifton, NJ)* **1728**, 169-190 (2018).
