# Supplementary material for: Lysine 68 acetylation directs MnSOD as a tetrameric detoxification complex versus a monomeric tumor promoter
Source: Nat Commun. 2019 Jun 3;10:2399. doi: 10.1038/s41467-019-10352-4 (PMC6546705; doi:10.1038/s41467-019-10352-4)
Supplement: Supplementary file 3 — Reporting Summary [file 41467_2019_10352_MOESM3_ESM.pdf]

nature research

Corresponding author(s): David Gius

Last updated by author(s): Apr 16, 2019

Reporting Summary

Nature Research wishes to improve the reproducibility of the work that we publish. This form provides structure for consistency and transparency in reporting. For further information on Nature Research policies, see Authors & Referees and the Editorial Policy Checklist.

Statistics

For all statistical analyses, confirm that the following items are present in the figure legend, table legend, main text, or Methods section.

n/a	Confirmed	
☐	☒	The exact sample size (n) for each experimental group/condition, given as a discrete number and unit of measurement
☐	☒	A statement on whether measurements were taken from distinct samples or whether the same sample was measured repeatedly
☐	☒	The statistical test(s) used AND whether they are one- or two-sided<br>*Only common tests should be described solely by name; describe more complex techniques in the Methods section.*
☒	☐	A description of all covariates tested
☒	☐	A description of any assumptions or corrections, such as tests of normality and adjustment for multiple comparisons
☐	☒	A full description of the statistical parameters including central tendency (e.g. means) or other basic estimates (e.g. regression coefficient) AND variation (e.g. standard deviation) or associated estimates of uncertainty (e.g. confidence intervals)
☒	☐	For null hypothesis testing, the test statistic (e.g. F, t, r) with confidence intervals, effect sizes, degrees of freedom and P value noted<br>*Give P values as exact values whenever suitable.*
☒	☐	For Bayesian analysis, information on the choice of priors and Markov chain Monte Carlo settings
☒	☐	For hierarchical and complex designs, identification of the appropriate level for tests and full reporting of outcomes
☒	☐	Estimates of effect sizes (e.g. Cohen's d, Pearson's r), indicating how they were calculated

Our web collection on statistics for biologists contains articles on many of the points above.

Software and code

Policy information about availability of computer code

Data collection	N/A
Data analysis	N/A

For manuscripts utilizing custom algorithms or software that are central to the research but not yet described in published literature, software must be made available to editors/reviewers. We strongly encourage code deposition in a community repository (e.g. GitHub). See the Nature Research guidelines for submitting code & software for further information.

Data

Policy information about availability of data

All manuscripts must include a data availability statement. This statement should provide the following information, where applicable:

- Accession codes, unique identifiers, or web links for publicly available datasets
- A list of figures that have associated raw data
- A description of any restrictions on data availability

This paragraph is located on page 32 of the manuscript.

The authors declare that all of the data supporting our findings of this study are available within the paper as well as the Supplementary Information file. In addition, all of the data that support the findings of this study are available on request from the corresponding author (DG). The MnSOD structure data has been deposited in the protein data bank (ID: 1PL4; DOI: 10.2210/pdb1PL4/pdb) (ref. PubMed: 14638684; DOI: 10.1074/jbc.M311310200).

Field-specific reporting

Please select the one below that is the best fit for your research. If you are not sure, read the appropriate sections before making your selection.

☒ Life sciences ☐ Behavioural & social sciences ☐ Ecological, evolutionary & environmental sciences

For a reference copy of the document with all sections, see nature.com/documents/nr-reporting-summary-flat.pdf

Life sciences study design

All studies must disclose on these points even when the disclosure is negative.

Sample size	Sample size for all mouse experiments were 10 mice per group that was chosen to address the concerns of reviewer number 3, who specifically asked for 10 mice per experimental group.
Data exclusions	No data were excluded from any analysis.
Replication	All experiments were done in triplicate, and text stating this is present in the figure legends.
Randomization	Not relevant to our study. There were no randomization experiments done in this manuscript.
Blinding	No blinded studies were necessary because we used a computer program to analyze the tissue microarray data, as shown in Figure 8. This text is in Figure legend 8: "The intensity of staining was quantified using HistoQuest software."

Reporting for specific materials, systems and methods

We require information from authors about some types of materials, experimental systems and methods used in many studies. Here, indicate whether each material, system or method listed is relevant to your study. If you are not sure if a list item applies to your research, read the appropriate section before selecting a response.

Materials & experimental systems

n/a	Involved in the study	
☐	☒	Antibodies
☐	☒	Eukaryotic cell lines
☒	☐	Palaeontology
☐	☒	Animals and other organisms
☒	☐	Human research participants
☒	☐	Clinical data

Methods

n/a	Involved in the study	
☒	☐	ChIP-seq
☒	☐	Flow cytometry
☒	☐	MRI-based neuroimaging

Antibodies

Antibodies used	Anti-Ki67: eBioscience<br>Cat#: 14-5698-82<br>Anti-MnSOD: Cell Signaling<br>Cat#: D3X8F<br>Anti-SIRT3: Cell Signaling<br>Cat#: D22A3<br>Anti-Actin: Cell Signaling<br>Cat#: 4970<br>Anti-IDH2: Cell Signaling<br>Cat#: 56439<br>Anti-OSCP: Santa Cruz Biotechnology<br>Cat#: sc-365162<br>Anti-Rabbit Secondary: Cell Signaling<br>Cat#: 7074<br>Mouse Secondary: Cell Signaling<br>Cat#: 7076<br>Anti-MnSOD K68-Ac: Ab-Cam<br>Cat#: Ab137037<br>Anti-MnSOD K122-Ac: Epitomics, Inc, Burlingame, CA, This company has been bought by Abcam, Inc.<br>Anti-OSCP K139-Ac: Epitomics, Inc, Burlingame, CA, This company has been bought by Abcam, Inc.<br>Anti-IDH2 K413-Ac: Epitomics, Inc, Burlingame, CA, This company has been bought by Abcam, Inc.
Validation	For all the commerically available antibodies, see the manufacturers' websites, which provides references and validations for their antibodies.

For anti-MnSOD-K68-Ac antibody text is provided in the figure legend of supplemental Figure s4, stating "A Flag-tagged MnSOD vector was transfected into HEK 293T cells with TSA (1 μM) and after 48 h Flag-MnSOD was IPed and samples were washed and incubated with purified SIRT3 protein without (lane 1) or with (lane 2) NAD (left panel). After 2 h mixtures were immunoblotted with an anti-MnSOD-K68-Ac antibody (Abcam, Inc). Identical experiments were done with the control non-acetylated peptide (middle panel) or the 13 amino acid K68-Ac peptide (right panel). Livers were harvested from isogenic, 2-month-old age-matched mice that were placed on an ad libitum (AL) or caloric restriction (CR) diet for 12 weeks. Mitochondrial extracts were isolated, separated, and subsequently blotted with antibodies to MnSOD, MnSOD-K68-Ac, SIRT3, and COXIV.".

For validation of the other anti-acetylated antibodies, text for sources of validation is provided in the figure legend of supplemental Figure s5, stating "The cell lysates were analyzed by immunoblotting with anti-MnSOD-K122-Ac (validated as a SIRT3 deacetylation target in Tao et al., 2010, Cancer Cell), anti-MnSOD, anti-OSCP-K139-Ac (validated as a SIRT3 deacetylation target in Tao et al., 2010, Cancer Cell), anti-OSCP, anti-IDH2K413-Ac (validated as a SIRT3 deacetylation target in Someya et al., 2010, Cancer Cell), anti-IDH2, and anti-actin.".

Eukaryotic cell lines

Policy information about cell lines

Cell line source(s)	ATCC
Authentication	HEK-293T, MCF7, T47D and NIH3T3 cells were obtained from ATCC in 2012, authenticated using STR profiling with CellCheck 9 Plus by IDEXX Bioresearch, and tested for mycoplasma using PlasmoTest™ - Mycoplasma Detection Kit (InvivoGen, Inc). Early passages of cells were frozen, and all cells were passaged for fewer than six months.
Mycoplasma contamination	All cell lines were tested for mycoplasma using PlasmoTest™ - Mycoplasma Detection Kit (InvivoGen, Inc).
Commonly misidentified lines (See ICLAC register)	None

Animals and other organisms

Policy information about studies involving animals; ARRIVE guidelines recommended for reporting animal research

Laboratory animals	Species:<br>- mice, athymic nude, nu/nu, female, 6-7 weeks, purchased from The Jackson Laboratory.
Wild animals	None
Field-collected samples	None
Ethics oversight	Northwestern University Institutional Animal Care and Use Committee (IACUC)

Note that full information on the approval of the study protocol must also be provided in the manuscript.
